# MYC copy gain, chromosomal instability and PI3K activation as potential markers of unfavourable outcome in trastuzumab-treated patients with metastatic breast cancer

**DOI:** 10.1186/s12967-016-0883-z

**Published:** 2016-05-17

**Authors:** Helen Gogas, Vassiliki Kotoula, Zoi Alexopoulou, Christos Christodoulou, Ioannis Kostopoulos, Mattheos Bobos, Georgia Raptou, Elpida Charalambous, Eleftheria Tsolaki, Ioannis Xanthakis, George Pentheroudakis, Angelos Koutras, Dimitrios Bafaloukos, Pavlos Papakostas, Gerasimos Aravantinos, Amanda Psyrri, Kalliopi Petraki, Konstantine T. Kalogeras, Dimitrios Pectasides, George Fountzilas

**Affiliations:** First Department of Medicine, Laiko General Hospital, National and Kapodistrian University of Athens School of Medicine, Athens, Greece; Department of Pathology, Aristotle University of Thessaloniki School of Medicine, Thessaloniki, Greece; Laboratory of Molecular Oncology, Hellenic Foundation for Cancer Research/Aristotle University of Thessaloniki School of Medicine, Thessaloniki, Greece; Department of Biostatistics, Health Data Specialists Ltd, Athens, Greece; Second Department of Medical Oncology, Metropolitan Hospital, Piraeus, Greece; Department of Medical Oncology, Papageorgiou Hospital, Aristotle University of Thessaloniki School of Medicine, Thessaloniki, Greece; Department of Medical Oncology, Ioannina University Hospital, Ioannina, Greece; Division of Oncology, Department of Medicine, University Hospital, University of Patras Medical School, Patras, Greece; First Department of Medical Oncology, Metropolitan Hospital, Piraeus, Greece; Oncology Unit, Hippokration Hospital, Athens, Greece; Second Department of Medical Oncology, Agii Anargiri Cancer Hospital, Athens, Greece; Division of Oncology, Second Department of Internal Medicine, Attikon University Hospital, Athens, Greece; Pathology Department, Metropolitan Hospital, Piraeus, Greece; Translational Research Section, Hellenic Cooperative Oncology Group, Data Office, Athens, Greece; Oncology Section, Second Department of Internal Medicine, Hippokration Hospital, Athens, Greece; Aristotle University of Thessaloniki, Thessaloniki, Greece

**Keywords:** Metastatic breast cancer, Trastuzumab, HER2, MYC, MET, Centromere copy number, FISH, qPCR, Amplification, TOP2A

## Abstract

**Background:**

There is an unmet need for more efficient patient stratification for receiving trastuzumab in the metastatic breast cancer (mBC) setting, since only part of such patients benefit from the addition of this agent to chemotherapy. The aim of this study was to investigate the prognostic value of biomarkers including MYC and MET in mBC patients treated with trastuzumab-based regimens.

**Methods:**

mBC patients, locally tested as HER2-positive, treated with trastuzumab and chemotherapy between 1998 and 2010 were evaluated. Paraffin tumors (n = 229) were retrospectively centrally assessed by immunohistochemistry (IHC) for HER2, ER, PgR and Ki67; fluorescence in situ hybridization (FISH) for HER2, TOP2A and centromere (CEN) 17, MYC and CEN8, MET and CEN7; qPCR for MYC, MET copy number (CN); and, for PI3K activation (PIK3CA mutations; PTEN and phospho-mTOR protein expression). Increased CEN CN was assessed based on normal cut-offs. Time to progression (TTP) and survival were evaluated from the initiation of trastuzumab as first line treatment.

**Results:**

Among all tumors, 90 were HER2-negative upon central testing (ambiguous HER2) and the rest were true HER2-positive. Further, 156 patients presented with mBC upon relapse of pre-treated disease (R-mBC) and 65 were diagnosed at stage IV (de novo mBC). Concordance between FISH and qPCR on gene CN status was fair for MYC (Kappa = 0.458) and absent for MET. The presence of MYC CN gain with qPCR and the absence of PI3K activation were infrequent events (7 and 8 % of evaluable tumors, respectively), while 41 % of tumors had increased CEN CN in one or more chromosomes, indicative of chromosomal instability. The most consistent finding in the entire cohort and in the above patient subgroups with respect to outcome was the unfavourable effect of MYC CN gain, which was retained upon multivariable analysis (e.g., survival in the entire cohort, HR 6.02; 95 % CI 2.67–13.6; p < 0.001). Further unfavourable prognosticators were increased CEN CN in one chromosome in R-mBC but not in de novo mBC (multivariable interaction p = 0.048), PI3K activation in R-mBC (multivariable p = 0.004) and increased Ki67 for patient TTP.

**Conclusions:**

MYC gene copies, centromere status and PI3K activation may adversely impact trastuzumab treated mBC patient outcome and seem worthy validating in larger series.

**Electronic supplementary material:**

The online version of this article (doi:10.1186/s12967-016-0883-z) contains supplementary material, which is available to authorized users.

## Background

The proto-oncogene HER2 (ERBB2) is amplified in approximately 20 % of breast cancers and is associated with a number of adverse prognostic factors, such as increased proliferative indices [1, 2], metastasis and recurrence. Trastuzumab, the first of a series of anti-HER2 agents, is a recombinant monoclonal antibody directed against the extracellular domain of the HER2 protein and has become the standard of care for patients with HER2-positive breast cancer, since it prolongs progression-free and overall survival in the adjuvant or metastatic settings [3, 4]. However, there is a large proportion of HER2-positive patients that do not gain clinical benefit from such agents either as single agents or in combination with chemotherapy [5], many of them presenting with up-front resistance [1, 6, 7]. Efforts are still ongoing for refining treatment strategies by incorporating different anti-HER2 agents, thus sparing both potential unnecessary toxicities associated with therapy and also potential treatment failures resulting from inappropriate treatment schedules or from treatments that negatively impinge upon survival [8]. For the identification of more efficient prognostic biomarkers in patients treated with trastuzumab, the effects of genes and proteins implicated in the HER2 signalling pathway(s) are usually studied on patient tumor material.

By-pass of signalling or de-novo resistance to targeted agents are important mechanisms that cancers utilize to fight our therapeutic armamentarium. Elucidation of de-novo or acquired mechanisms of resistance to several agents requires the same diligence for finding the targetable biomarker, as we further understand the heterogeneity of tumors. HER2 is a member of the HER tyrosine kinase family expressed in virtually all cancer types. Interaction of the HER-signalling pathway(s) with the PI3K/AKT signalling pathway that plays a role in cell growth, metabolism, cell survival and oncogenesis [9, 10] is well acknowledged. MET over-expression or gene gain on chromosome 7q31 has been reported in a variety of tumors including breast cancers, correlating with poor prognosis [11]. MET activates many of the downstream pathway members of HER2, including PI3K, AKT, PLCg and RAS-MAPK, and as such may act as a negative indicator of trastuzumab efficacy.

MYC, a proto-oncogene located at 8q24.1, plays a central role in proliferation and malignant transformation. The gene encodes 3 different transcription factors that play a role in normal cellular function, replication, proliferation and apoptosis [12, 13]. Aberrations in MYC have been reported to play a key role as potent activators of malignant transformation and progression in several types of human malignancies [14, 15]. In breast cancer its amplification and/or overexpression is consistently observed in more aggressive ER-negative disease, correlating with poor prognosis and distant metastases [16–18]. Depending on the technique used [19], several inconsistent correlations with clinicopathological factors have been reported, as has the frequency of overexpression ranging from 1–95 %. FISH has been considered to be the optimal method for assessing MYC gene status, as it includes a centromeric probe that appears necessary to differentiate between polysomy and gene amplification [20].

In the present retrospective translational research study, we sought to investigate the effect of MET and MYC status on the outcome of trastuzumab treated patients with mBC in a cohort previously analyzed by our group for PIK3CA and PTEN, as well as HER2 and TOP2A status [21, 22]. Herein, MET and MYC were investigated with four different methods at the gene (chromosome), mRNA and protein levels. In addition, the number of centromere copies for the three chromosomes harbouring the genes studied (chr17, chr7, and chr8) was also analyzed. Finally, because of the central role of the PI3K pathways in the maintenance of HER2-positive tumors, MET and MYC status were assessed in combination with PI3K/AKT/mTOR parameters.

## Methods

### Patients and tumor tissue material

The study was performed on formalin-fixed paraffin-embedded (FFPE) tumor tissue material collected from patients with mBC that were treated with trastuzumab-based combinations between December 1998 and January 2010 in HeCOG affiliated clinical centers, as described previously [21, 22]. Eligibility criteria for case analysis in the present study were, histologically confirmed mBC; adequacy of clinical data on patient’s history, demographics, tumor characteristics, treatment details (drug dosage, schedule of administration, adverse events) and clinical outcome (patients lost to follow-up were excluded); availability of adequate FFPE tumor tissue for biological marker evaluation; trastuzumab-based treatment for metastatic disease; and, no adjuvant treatment with trastuzumab. The translational research protocol was approved by the Bioethics Committee of the Aristotle University of Thessaloniki School of Medicine (#4283; January 14, 2008) under the general title ‘‘Investigation of major mechanisms of resistance to treatment with trastuzumab in patients with metastatic breast cancer’’. All patients included in the study after 2005, provided written informed consent for the provision of biological material for future research studies, before receiving any treatment. Waiver of consent was obtained from the Bioethics Committee for patients included in the study before 2005. Patients presented with mBC either upon relapse of previously treated disease (R-mBC) or de novo, without previous history of breast cancer. However, tissue material from the primary tumors was examined in most cases.

### FFPE tissue processing

Patients had received trastuzumab based on HER2 assessment in local pathology laboratories. However, because of the broad period of patient recruitment during which ER, PgR and HER2 guidelines for breast tumor typing and patient stratification for trastuzumab treatment were repeatedly modified, all tumors were re-evaluated centrally for these basic breast cancer typing parameters, in the laboratory of Molecular Oncology. ER, PgR and HER2 were evaluated according to ASCO/CAP guidelines as previously published [21, 22].

In total, 229 cases meeting the above eligibility criteria for patients and tissues were examined. Corresponding tumors were histologically evaluated on hematoxylin & eosin (H&E) sections for tumor presence and marked for the most tumor dense areas. Tumor cell content (TCC) was assessed as the ratio of cancer cells vs. non-cancer cells in these areas, which were used for manual macrodissection for DNA/RNA extraction and, upon a second H&E evaluation, for obtaining cores for tissue microarray (TMA) construction, in this order. Manual macrodissection was performed on 10 micron thick unstained sections and processed for dual nucleic acid extraction with silica-coated magnetic beads (Versant Tissue Preparation Reagents, Siemens Healthcare Diagnostics, Tarrytown, NY, USA), according to the manufacturer’s instructions. Based on the abundance of tumor tissue on blocks and the availability of thick sections, extracts were divided into two aliquots for storage at −20 °C until use. DNase I was added to one aliquot per sample for removing DNA and ensuring the presence of pure RNA for gene expression analyses. TMAs were used for all in situ methods, i.e., immunohistochemistry (IHC) and fluorescent in situ hybridization (FISH). These methods were performed on 3 and 5 micron thick TMA sections, respectively. Seventeen TMA blocks were constructed with a manual tissue microarrayer (Beecher Instruments, Sun Prairie, WI, USA), using 2 cores per tumor, each 1.5 mm in diameter, along with orientation and IHC control sample cores. For cases with low tumor tissue availability, inclusion of tumor tissue in TMAs was prioritized over DNA/RNA extraction. Thus, all 229 tumors were included in TMAs, while RNA was prepared from 207 tumors and DNA from 182. TCC was ≥30 % in 93 % of these cases.

### IHC

Except for the above mentioned ER, PgR and HER2, Ki67 (clone MIB-1, Dako, Glostrup, DK), p-mTOR^Ser2448^ (clone 49F9, CST, Danvers, MA, USA) and PTEN (clone 6H2.1, Dako) were also examined with IHC, as previously described [23–25]. Ki67 classification as high (≥14 %) and low (<14 %) was applied for distinguishing the centrally evaluated HER2-negative tumors as Luminal A and B [24]. PTEN protein expression (cytoplasmic, nuclear or both) was evaluated according to a staining intensity scale from 0 (negative, no staining) to 3 (intense staining). Tumors with PTEN IHC scores of 0 or 1 were considered to have PTEN loss. The phosphorylated form of mTOR at Ser2448, was defined as positive if at least mild cytoplasmic staining was detected in >1 % of tumor cells. In addition, c-Myc protein expression was assessed with a monoclonal antibody (clone 9E10, Dako, 1:300 dilution). For c-Myc protein, immunoreactive score (IRS) was calculated based on the intensity of positive staining and number of stained cells. Intensity scores of 0 (no staining), 1 (mild), 2 (moderate), 3 (high intensity) and percentage scores were assigned as 1 = 1–25 %; 2 = 26–50 %; 3 = 51–75 %; and 4 = 76–100 %. The IRS score ranged from 0 to 12 and classified tumors into four categories for negative (score 0–1); low (2–3); intermediate (4–5); and high (6–12) c-Myc protein expression.

The evaluation of all IHC was conducted by experienced breast cancer pathologists (I.K. and M.B.), blinded as to the patients’ clinical characteristics and survival data.

### FISH

FISH for the investigation of MET and MYC gene status was performed with the Zyto*Light* SPEC MET/CEN7 (Z-2087-200, ZytoVision Bremerhaven, Germany) and LSI CMYC SpectrumOrange-labeled probe combined with CEP8 SpectrumGreen-labeled probe (both from Abbott Molecular, Abbott Park, IL, USA). CEP8 detects the centromeric region of the corresponding chromosome, hence it will be called CEN8 for uniformity. The method was performed according to the manufacturer’s protocol with minor modifications. Sixty non-overlapping nuclei from the invasive front of the tumor were randomly selected, according to morphological criteria using DAPI staining, and scored (E.T and M.B) for both green (MET and CEP8) and orange (MYC and CEN7) signals. For all probes, sequential (5 or more planes at 0.85–1.0 μm) digital images were captured using the Plan Apo VC 9100/1.40 objective (Nikon, Kanagawa, Japan) using specific filters for each probe. The resulting images were reconstructed using specifically developed software for cytogenetics (XCyto-Gen, Alphelys, Plaisir, France). Normal breast tissue specimens (n = 20) were used as a control of the FISH assays. All primary image data of the TMA and whole tumor sections have been digitally scanned and made publicly available at: http://www.hecog-images.gr/index.php?dir=/home/gkatak/public_html/MET/CEN7/FISH_TRANSTUZUMAB and http://www.hecog-images.gr/index.php?dir=/home/gkatak/public_html/C-MYC/CEN8/FISH_TRASTUZUMAB, for MET and MYC, respectively.

MET amplification with FISH was defined as MET/CEN7 ratio ≥2.0 and/or ≥4 average MET copies per nucleus. MYC amplification was defined as MYC/CEP8 ratio ≥2.0 [26] and as MYC/CEN8 ratio ≥2.2 and/or ≥5 copies per nucleus [27]. Except for MET and MYC, TOP2A gene status data as obtained with the triple assay used for HER2 gene assessment were also used with average ratio TOP2A/CEN17 ratio ≥2.0 and/or >4 TOP2A gene copies per nucleus as cut-off for amplification [28].

In addition, “polysomy” was investigated for all assessed chromosomes in the FISH assays, by classifying the number of observed centromere signals (CEN17 with the triplex assay; CEN7 for MET and CEP8 for MYC) based on counts in normal nuclei. Clearly, because only the centromeres and the specific gene loci were assessed per chromosome, observation of >2 centromere signals does not necessarily correspond to chromosome polysomy or altered ploidy. Thus, we refer to increased CEN copies for this condition throughout the manuscript. Cut-offs for increased CEN copies were assessed on 20 normal breast specimens and were calculated as normal mean CEN signal counts plus 3XSD, as previously suggested [29]. Thus, increased CEN17 copies were called for >3.22; CEN7 for >3.36; and CEN8 for >3.13 mean signal counts per tumor.

### DNA analyses for PIK3CA mutations and MET and MYC copy numbers

Mutation testing for hotspot PIK3CA mutations E542K and E545K (coding exon 9) and H1047R (coding exon 20) was accomplished with custom Taqman-MGB-SNP genotyping assays (duplex qPCR for the detection of control DNA and mutant target in the same reaction, as previously described [22].

Copy number (CN) analysis for the MET and MYC genes was implemented with real time PCR (qPCR) in 180 DNA samples, with premade CNV assays (Life Technologies/Applied Biosystems, Paisley, UK). For each gene, two genomic regions were targeted in order to increase analysis specificity and sensitivity. Assay ID, Genbank reference, location within gene and amplicon size for the genes analyzed were: MET on chromosome 7q31 (Hs02633538_cn; NM_000245.2, NM_001127500.1; exon 8; 80 bp/Hs05005398_cn; NM_000245.2, NM_001127500.1; intron 9; 107 bp); and MYC on chromosome 8q24.21 (Hs00834648_cn; NM_002467.4; exon 2; 106 bp/Hs00292858_cn; NM_002467.4; exon 3; 112 bp). The method involves duplex reactions, for the target gene, TaqMan® minor groove binding (MGB) probes, FAM™ labeled; for the reference gene, Taqman VIC®-TAMRA™ labeled probes, both assays with unlimited primers. TaqMan® copy number reference assay RNase P was used as endogenous reference. Reactions (10 μl) were run in quadruplicates in an ABI7900HT system in 384-well plates under default conditions. Three peripheral blood DNA samples from non-cancer patients were included in each run as calibrator samples, along with no-template controls (NTC). Results were obtained automatically with the CopyCaller™ Software v2.0 as predicted CN, in comparison to averaged calibrator values upon setting the evaluation threshold at Cp (crossing point) = 32 for the reference RNaseP in each reaction. The ΔΔCT method was employed to estimate the CT difference (ΔCT) between target and reference sequence in tumor samples as compared to the corresponding values of the calibrator samples. Based on the Cp = 32 eligibility cut-off, out of 180 samples, 158 were informative for MET and 168 for MYC copy number assessment (87.8 and 93.3 %, respectively).

MET and MYC CN were classified as no-gain for quadruplicate average CN ≤ 2.5 and as gain for average CN > 2.5. The cut-off 2.5 was chosen arbitrarily in order to exclude DNA replication. Both assays per gene had to be informative for the tumor evaluation. In cases with discrepant results between the two assays (no-gain and gain for the same tumor), gain was called for that particular gene.

### Relative MET and MYC gene expression

cDNA synthesis was applied on the 207 RNA samples described above, with random primers and SuperScript® III Reverse Transcriptase (Invitrogen™, Paisley, UK; cat. no. 48190011 and 18080044, respectively), according to the manufacturer’s instructions. cDNAs were assessed in duplicate 10 μl reactions in 384-well plates with qPCR in an ABI7900HT system for 45 cycles of amplification (default conditions). The following exon-spanning premade Taqman-MGB assays (Applied Biosystems/Life Technologies) were selected for the transcripts under investigation (data in parentheses refer to assay ID; Genbank reference; amplicon location; size): MET (Hs00179845_m1; NM_001127500.1, NM_000245.2; exons 10–11; 81 bp); MYC (Hs00153408_m1; NM_002467.4; exons 2–3; 107 bp). A Taqman-MGB expression assay targeting β-glucuronidase (GUSB) mRNA (Hs00939627_m1; NM_000181.3; exons 8–9; 96 bp) was used for the assessment of relative quantification. GUSB was selected as the endogenous reference since, among the widely used housekeeping genes, it does not seem to be represented in pseudogenes. In addition, GUSB has been independently identified as one among the best preserved mRNA targets in FFPE tissues [30, 31]. A commercially available reference RNA derived from multiple transformed cell lines (TaqMan® Control Total RNA, cat. no 4307281, Applied Biosystems) was applied in multiple positions in each run as positive control and for inter-run evaluation of PCR assay efficiency. To obtain linear Relative Quantification (RQ) values, relative expression was assessed as (40-dCT), whereby dCT (or delta Cycle Threshold, equivalent to Cq in MIQE guidelines) was calculated as (average target CT)—(average GUSB CT) from all eligible measurements under the sane reading threshold. Samples were considered eligible for GUSB CT <36 and deltaRQ for each duplicate pair (intra-run variation) of <0.8. Inter-run RQ values for the reference RNA were <1 for both assays. By using these criteria, 104 tumor samples were informative for MET and 140 for MYC relative mRNA expression.

### Statistical analysis

All examined biomarkers are presented as category frequencies and corresponding percentages, while associations with HER2 status were examined using the Chi square or Fisher’s exact tests, where appropriate. For continuous mRNA RQ values the median was used for classifying low vs. high expression. Comparisons between MET and MYC FISH and qPCR CN were performed by calculating the Cohen’s Kappa measure of agreement. Binary variable clustering was also performed for: CEN17/CEN7/CEN8 polysomy as 0, 1 and >1, corresponding to the number of chromosomes with alterations.

Central IHC/FISH typing for the 229 available tumors revealed 139 HER2-positive and 90 HER2-negative cases. The rate of discordance (39.7 %) between local and central typing was excessively high and appears to be due: to the different criteria used and the experience of pathologists over 13 years in calling HER2-positive tumors; to pre-analytical and analytical conditions of the methods; and, also, to tumor heterogeneity with respect to HER2 over-expression/amplification status [32, 33]. HER2 heterogeneity should definitely be considered as an issue for the observed discrepancy in this study, although unpredictable, since the retrospective identification of different tumor blocks that were used for local and central testing was impossible. In order to cover the HER2 status discrepancy, all parameters were analyzed in the entire cohort and separately in the centrally labelled HER2-positive and negative groups. Table 1 includes selected demographic and tumor characteristics in the entire cohort.

**Table 1:** Patient characteristics in the entire cohort and according to central HER2 status

	Entire cohort		HER2-negative		HER2-positive		p value
N (%)	229		90 (39.3 %)		139 (60.7 %)		
Age (years)							
Median (range)	57 (28–95)		59 (32–79)		55 (28–95)		*0.035*

	N	%	N	%	N	%	p value
Menopausal status							
Pre	71	31	28	31.1	43	30.9	0.98
Post	158	69	62	68.9	96	69.1	
Performance status (N = 225)							
0	164	72.9	63	70.8	101	74.3	0.38
1	47	20.9	18	20.2	29	21.3	
2–3	14	6.2	8	9	6	4.4	
History (N patients with available data)							
Adjuvant CT (N = 228)	133	58.3	55	61.8	78	56.1	0.40
Adjuvant CT anthracycline (N = 227)	90	39.6	29	33	61	43.9	0.10
Adjuvant CT taxanes (N = 227)	55	24.2	17	19.3	38	27.3	0.17
Adjuvant RT (N = 224)	87	38.8	35	39.8	52	38.2	0.82
Adjuvant HT (N = 227)	108	47.6	45	51.1	63	46.3	0.48
Line of treatment							
1st line	192	83.8	68	75.6	124	89.2	
2nd line	31	13.5	18	20.0	13	9.4	
3rd line	2	0.9	2	2.2	0	0.0	
4th line	2	0.9	1	1.1	1	0.7	
5th line	1	0.4	0	0.0	1	0.7	
6th line	0	0.0	0	0.0	0	0.0	
7th line	1	0.4	1	1.1	0	0.0	
Histological grade (N = 214)							
I–II	94	43.9	40	47.6	54	41.5	0.38
III	120	56.1	44	52.4	76	58.5	
Metastatic sites							
Locoregional	73	31.9	28	31.1	45	32.4	0.84
Distant	203	88.6	83	92.2	120	86.3	0.17
Bones	98	42.8	41	45.6	57	41.3	0.53
Visceral	155	67.7	60	66.7	95	68.3	0.79
Number of metastatic sites							
<3	166	72.4	65	72.2	101	72.7	0.87
≥3	63	27.6	25	27.8	38	27.3	

*CT* chemotherapy, *RT* radiotherapy

Significant p values are shown in italics

Data on selected patient and tumor characteristics, previous and subsequent lines of treatment, disease progression events and survival were obtained from medical records and were entered into a central database. Follow-up information for all patients was updated in July 2015. Since not all patients had metastatic disease upon first diagnosis, the cohort was divided into two groups: (a) patients who presented with relapse from previously treated disease (R-mBC); and (b) patients who presented with metastatic breast cancer as first diagnosis (de novo mBC).

Patients received T in the 1st line of chemotherapy for metastatic disease and follow-up was updated in June 2015. Time to progression (TTP) was defined as the time from T-treatment initiation in the 1st line treatment (with or without concurrent chemo/hormonal therapy) to the date of documented disease progression. Survival was measured from initiation of T-treatment in the patients receiving the drug as a 1st line treatment to the date of death. Patients alive were censored at the date of the last follow-up contact. Survival probabilities were estimated by the Kaplan–Meier method. For the univariate and multivariate analyses, Cox proportional hazards models were used. Univariate analyses were performed in the centrally identified HER2-positive and HER2-negative groups and, in the R-mBC and de novo mBC disease groups.

**Fig. 1:**
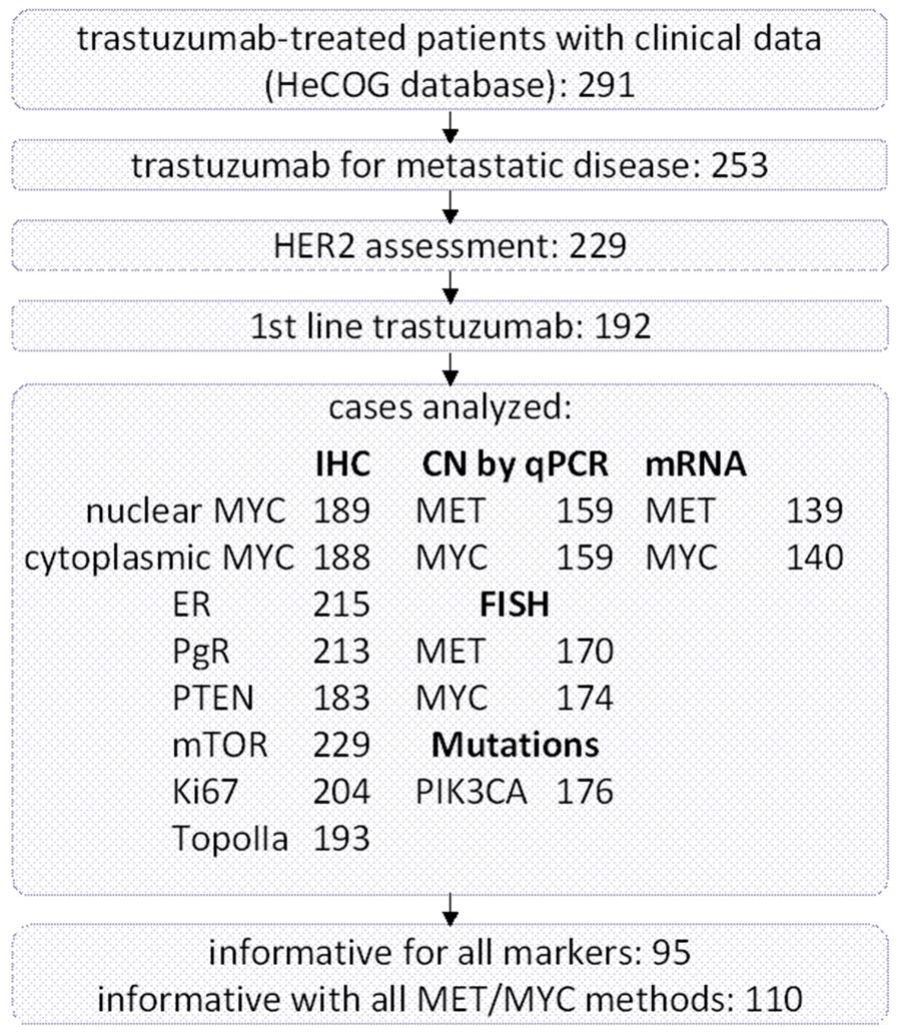
Remark diagram for the biological material tested

All tests were two-sided at a = 0.05 level of significance. No adjustment for multiple comparisons was performed. Multivariate analysis was performed in the entire cohort adjusted for HER2 status. Model choice was performed using backward selection with a removal criterion = 0.15, including in the initial step clinical parameters such as: age, menopausal status, performance status, receptor status (ER/PgR), Ki67 continuous expression, number of metastatic sites, presentation of distant metastasis, presentation of disease (R-mBC vs. de novo-mBC) and HER2-status. The statistical analysis complied with the reporting recommendations for tumor marker prognostic studies (REMARK) [34] and was performed using the SAS software (SAS for Windows, version 9.3, SAS Institute Inc., Cary, NC, USA). The number of informative cases along with all markers and applied methods are shown in Fig. 1.

## Results

### Association of markers with HER2 status and subtypes

Clinicopathological characteristics were similar in central HER2-positive vs. HER2-negative tumors (Table 1) and in tumors from patients with R-mBC vs. de novo-mBC (Additional file 1: Table S1) except for age and for the number of metastatic sites according to disease presentation.

**Fig. 2:**
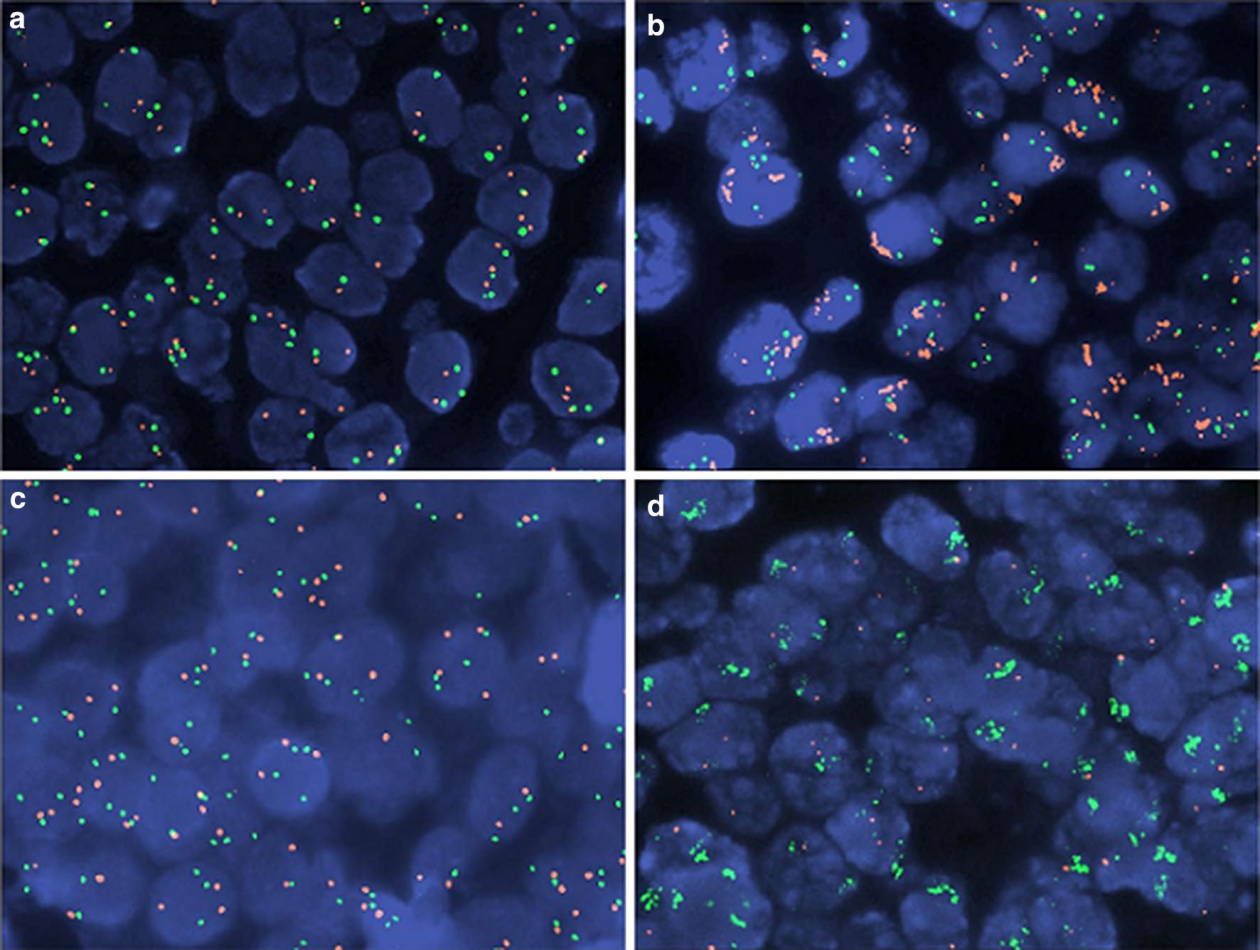
Examples of MYC and MET status by FISH. **a** Normal MYC; **b** amplified MYC; **c** normal MET; **d** one of four amplified MET tumors

In the entire cohort, 31 and 4 tumors were called MYC and MET amplified with FISH, respectively. Representative examples are shown in Fig. 2. In comparison, 15 and 40 tumors had MYC and MET copy gains with qPCR, respectively. Conceivably, low concordance was noticed for MYC status with qPCR and FISH (Kappa 0.458 for qPCR vs FISH ratio; Kappa 0.416 for qPCR vs. FISH copies), while no concordance was revealed for MET, respectively. Similarly, no association was noticed between MET and MYC gene status and mRNA expression levels (high/low at any percentile) and c-Myc protein expression according to MYC gene status. c-Myc protein expression was more often absent or nuclear in centrally assessed HER2-positive as compared to negative tumors (Table 2), and so was high MET and MYC mRNA expression. The 4 cases with MET amplification, as assessed with FISH, and the majority of MYC amplified tumors were also HER2-positive. MET and MYC CN status by qPCR did not differ between HER2-negative and positive tumors.

**Table 2:** Marker associations according to central HER2 status

	HER2-negative		HER2-positive		p value
Ki67 (%)					
Median (range)	38 (1–90)		40 (1–90)		0.12
HER2 copies (FISH)					
Median (range)	2 (1–9)		13 (2–39)		<*0.001*
Mean (±SD)	3 (1)		14 (8)		
HER2 ratio (FISH)					
Median (range)	1 (1–8)		5 (1–28)		<*0.001*
Mean (±SD)	1 (1)		7 (4)		
CEN17					
Median (range)	2 (1–4)		2 (1–4)		*0.006*
Mean (±SD)	2 (1)		2 (1)		
CEN8					
Median (range)	3 (1–9)		2 (1–5)		0.80
Mean (±SD)	3 (1)		3 (1)		
CEN7					
Median (range)	2 (1–4)		2 (1–5)		0.50
Mean (±SD)	2 (1)		2 (1)		

	N	%	N	%	p value
IHC, categorical					
ER (N = 229)					
Negative	16	17.8	60	43.2	<*0.001*
Positive	74	82.2	79	56.8	
PgR (N = 229)					
Negative	32	35.6	89	64	<*0.001*
Positive	58	64.4	50	36	
mTOR (N = 229)					
Negative	36	40	48	34.5	0.40
Positive	54	60	91	65.5	
PTEN (N = 183)					
Negative (0–1)	47	65.3	55	49.5	*0.036*
Positive (2–3)	25	34.7	56	50.5	
c-MYC (N = 188)					
Both negative	19	24.4	41	37.3	*0.011*
Both positive	27	34.6	31	28.2	
Cytoplasmic only	28	35.9	22	20	
Nuclear only	4	5.1	16	14.5	
Mutations					
PIK3CA (N = 176)					
WT	49	69	88	83.3	*0.020*
Mutant	22	31	17	16.2	
Gene CN by qPCR					
MET (N = 159)					
No gain	42	71.2	77	77	0.41
Gain	17	28.8	23	23	
MYC (N = 159)					
No gain	54	90	90	90.9	0.85
Gain	6	10	9	9.1	
Gene status by FISH					
MET (N = 164)					
Non-amplified	56	100	104	96.3	0.15
Amplified			4	3.7	
MYC (N = 173)					
Non-amplified	56	88.9	86	78.2	0.077
Amplified	7	11.1	24	21.8	
TOP2A (N = 226)					
Non-amplified	82	93.2	74	53.6	<*0.001*
Amplified	6	6.8	64	46.4	
CEN17 (N = 226)					
Normal	82	93.2	119	86.2	0.10
Increased	6	6.8	19	13.8	
CEN7 (N = 170)					
Normal	54	90	101	91.8	0.69
Increased	6	10	9	8.2	
CEN8 (N = 174)					
Normal	47	74.6	79	71.2	0.63
Increased	16	25.4	32	28.8	
CEN profiles (N = 170)					
0 chromosomes	40	66.7	60	54.5	0.21
1 chromosome	13	21.7	41	37.3	
2 chromosomes	6	10	8	7.3	
3 chromosomes	1	1.7	1	0.9	
mRNA					
MET (N = 139)					
Low	36	63.2	36	43.9	*0.025*
High (≥50th perc.)	21	36.8	46	56.1	
MYC (N = 140)					
Low	36	63.2	37	44.6	*0.031*
High (≥50th perc.)	21	36.8	46	55.4	

Significant p values are shown in italics

In comparison to centrally HER2-negative tumors (Table 2), centrally HER2-positive tumors were more often hormone receptor negative but positive for PTEN protein expression; at the genomic level, absence of PIK3CA mutations but presence of TOP2A amplification were also more common in these tumors. Ki67 labelling and p-mTOR protein expression did not differ between HER2-negative and positive tumors. PI3 K activation, reflected by PIK3CA mutations, PTEN protein absence and p-mTOR positivity, was observed in 197 (92.9 %) cases, 78 (95.1 %) being HER2-negative and 119 (91.5 %) HER2-positive patients.

The 139 centrally HER2-positive tumors were further examined as Luminal-HER2 and HER2-Enriched (Additional file 1: Table S2), based on hormone receptor presence and absence, respectively. High MET mRNA was more often encountered in HER2-Enriched tumors; all other markers did not differ between these two subtypes. Similarly, all markers examined were equally represented in R-mBC and de novo mBC (Additional file 1: Table S3).

Tumors with MYC amplification (FISH) or gain (qPCR CN) more frequently had nodal metastases (e.g., 8/14 tumors (57 %) with MYC gain vs. 27/110 (19 %) with no gain had positive nodes; p = 0.002), while tumors with normal MET more often had visceral metastases (53 % with qPCR MET CN gain vs. 78 % with normal MET; p = 0.004).

Finally, as described above, the status of chromosome 7, 8 and 17 centromeric regions was inferred from the centromere signal counts (CEN) in the FISH assays. A higher than normal copy number of CEN8 was associated with higher Ki67, as compared to tumors with normal CEN8 (Ki67 labelling mean ± SD of 57.6 ± 20.0 vs. 38.3 ± 20.2, respectively; Mann–Whitney, p < 0.001). CEN7 and CEN17 copies were not associated with any of the examined markers. Except for increased CEN17 copies that were associated with TOP2A amplification (p = 0.006), aberrant CEN7 and CEN8 copies did not respectively coincide with MET and MYC amplification or copy gain.

### Association of markers with clinical outcome

Median follow-up for all 1st line treated patients was 109.6, 107.8 months in the HER2-negative and 118.0 months in the HER2-positive group. The median TTP of the entire population was 14.5 months (95 % CI 11.7–17.8), 13.7 months (95 % CI 8.9–16.3) in the HER2-negative and 16.2 months (95 % CI 12.6–21.5) in the HER2-positive group. Of the 192 1st line treated patients, 146 (76.0 %) died. The median overall survival was 42.4 months (95 % CI 36.9–50.7). HER2-positive patients had numerically longer survival than HER2-negative patients (median survival 49.7 vs. 38.1 months, respectively, p = 0.14).

**Fig. 3:**
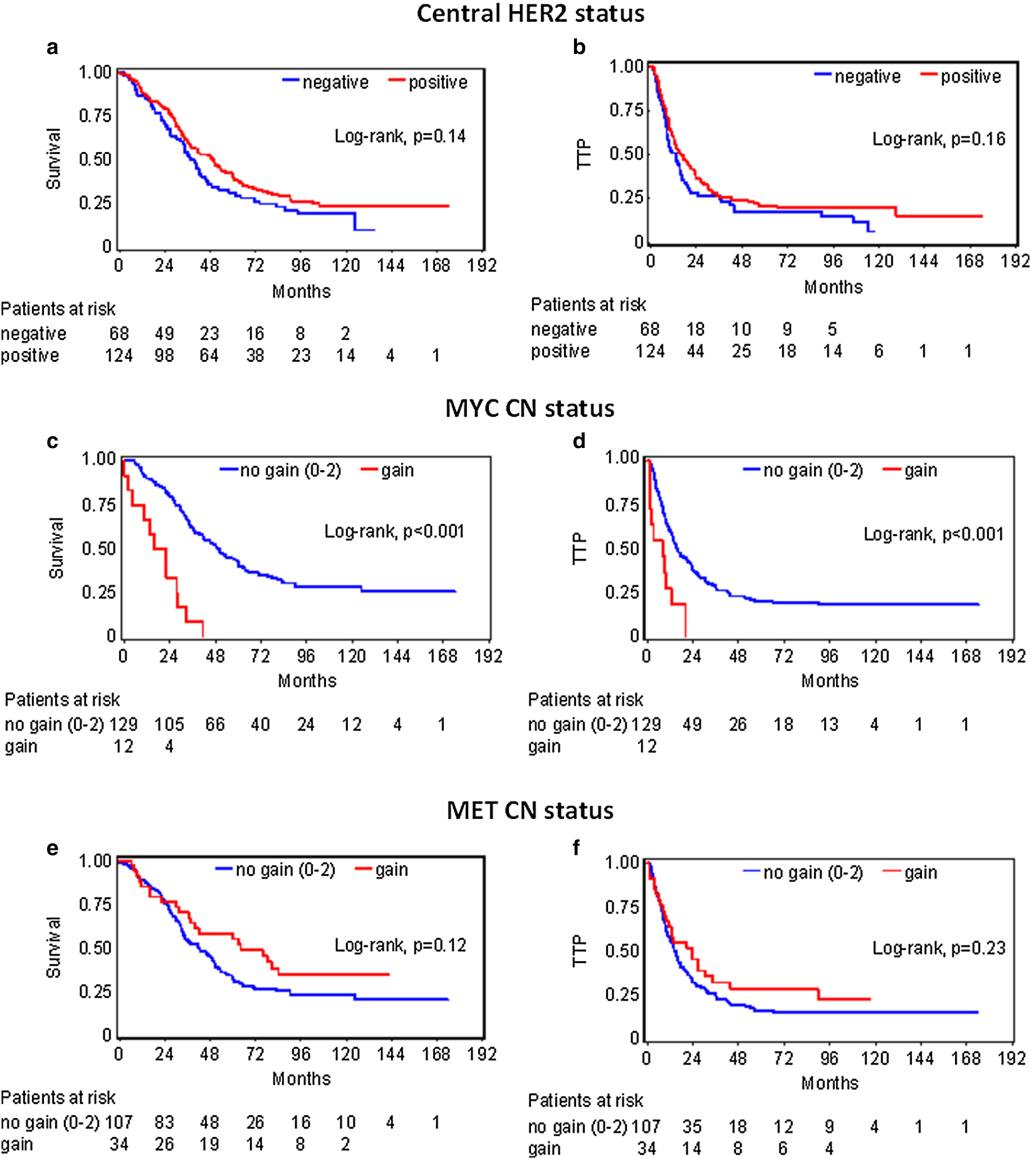
Patient outcome according to central HER2 status, MYC and MET copy numbers (CN). CN was assessed by qPCR. **a**, **c**, **e** survival; **b**, **d**, **f** time to progression

The prognostic significance of the examined markers upon univariate Cox analysis are shown in Additional file 1: Table S4 for the entire cohort. As mentioned, all mBC patients received trastuzumab, although 39 % of the tumors were found to be HER2-negative upon central evaluation. However, the central HER2-status did not significantly affect prognosis in this trastuzumab-treated cohort (Fig. 3a, b). Among typical clinicopathological parameters, worse PS and increased number of metastatic sites were associated with shorter survival, while PIK3CA mutations conferred shorter TTP. Increased MYC CN, as assessed by qPCR but not by FISH, was associated with increased risk for disease progression and death (Fig. 3c, d); increased CEN8 copies were also unfavourable for survival. By contrast, decreased risk for disease progression and death was conferred by PgR and PTEN protein expression and p-mTOR positivity, and also by TOP2A gene amplification. MET CN by qPCR (Fig. 3e, f) or by FISH, as well as CEN7 and CEN17 aberrations as single markers did not affect patient outcome.

When the cohort was split into central HER2-negative and HER2-positive, the majority of the above markers maintained their prognostic significance for survival (Additional file 1: Table S5) and TTP (Additional file 1: Table S6) in the same direction, but mostly in either group, HER2-negative or HER2-positive. As an exception, MYC CN gain was unfavourable in both groups with respect to survival. Interestingly, MET CN gain was favourable in the HER2-negative group only, for both TTP and survival. MYC CN gain retained its unfavourable prognostic significance for survival (Additional file 1: Table S7) and TTP (Additional file 1: Table S8) in both R-mBC and de novo-mBC patients, while TOP2A amplification was favourable in the absence of increased CEN17 copies in the R-mBC patients only.

**Fig. 4:**
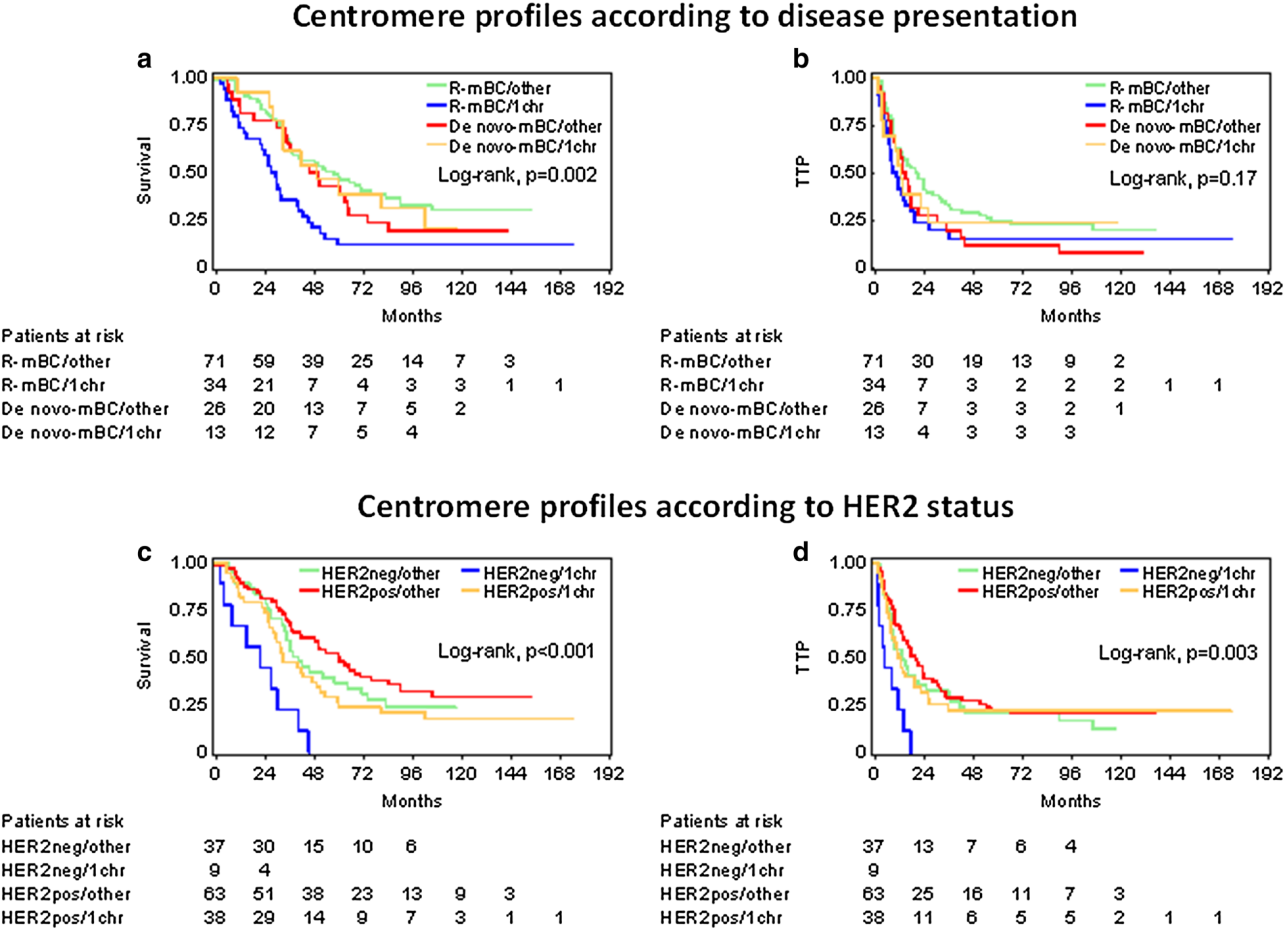
Effect of centromere profiles on the outcome of patients with metastatic breast cancer (mBC). In **a**, **b**, patients were distinguished in those who relapsed after adjuvant treatment (R-mBC), and those who presented with metastatic disease at first diagnosis (de novo mBC). In **c**, **d**, patients were grouped for centrally HER2-positive and HER2-negative disease. **a**, **c** Survival; **b**, **d** time to progression. *1chr* increased CEN copies in 1 chromosome only; *other* normal CEN or increased copies for >1 CEN

Profiling of aberrations in all three studied chromosome CEN revealed 100 tumors without any aberration, 54 with aberrant CEN probe signals in one, 14 in two and 2 in all three chromosomes. Increased CEN8 copies were identified in 48 tumors (Table 2) and were prevalent in the above CEN aberration profiles. For example, the group with 1 aberrant CEN included 34 patients with CEN8, 15 with CEN17 and 5 with CEN7 increased copies. Interestingly, patients with no aberrations and patients with two or three aberrant chromosomes in their tumors clustered together with respect to outcome. Thus, when using CEN patterns as a binary variable, patients bearing tumors with 1 aberrant chromosome fared worse than those with no or with more than 1 such alterations, particularly with respect to survival in the entire cohort (HR 1.72, 95 % CI 1.16–2.54, Wald’s p = 0.007, log-rank p = 0.006). The effect of these CEN profiles seemed specific for patients with R-mBC (for TTP, HR 1.62, 95 % CI 1.02–2.57, Wald’s p = 0.040; for survival, HR 2.39, 95 % CI 1.50–3.80, p < 0.001) (Fig. 4a, b). The interaction between CEN profiles and disease presentation was significant for patient survival (interaction p = 0.025; Additional file 1: Table S9); the presence of 1 aberrant chromosome was unfavourable for patients with R-mBC (HR 2.39, 95 % CI 1.50–3.80), but the same alteration was favourable among patients with de novo-mBC (HR 0.49, 95 % CI 0.24–1.00). When stratifying patients for central HER2 positivity, 1 aberrant chromosome conferred worse prognosis as compared to all other CEN profiles. The effect seemed more specific for patients with centrally evaluated HER2-negative tumors; such patients bearing tumors with 1 aberrant chromosome had the worst outcome (for TTP, HR 3.08, 95 % CI 1.45–6.56, Wald’s p = 0.004; for survival, HR 3.60, 95 % CI 1.67–7.76, p = 0.001) (Fig. 4c, d). The interaction between central HER2-status and CEN patterns (Additional file 1: Table S9) was significant for both disease progression and death (interaction p = 0.041 and 0.079, respectively). Among patients with tumors with 1 aberrant chromosome, those with centrally HER2-positive fared better than those with HER2-negative tumors. This alteration was an unfavourable prognosticator for TTP in HER2-negative but not in HER2-positive tumors. No difference was observed for CEN profiles on patient outcome with respect to HER2 subtypes (luminal and enriched).

Multivariate analysis for the parameters described was conducted in the entire cohort, in the central HER2-positive and HER2-negative groups, as well as in the R- and de novo mBC patient groups (Table 3 for survival and Additional file 1: Table S10 for TTP). ER/PgR positivity was the most consistent independent favourable prognosticator in the entire cohort, and separately in R-mBC and in de novo mBC patients. MYC CN independently predicted for worse TTP and survival in the entire cohort, in patients with centrally HER2-positive tumors and in patients with R-mBC. Further, in the entire cohort of patients treated with trastuzumab the interaction of CEN profiles with disease presentation was significant for survival, while the interaction with HER2 subtypes was significant for TTP. In this context, good patient performance status and TOP2A amplification also independently predicted for longer survival. Central HER2 positivity did not affect patient outcome.

**Table 3:** Multivariate Cox regression analysis for patient survival

Entire cohort (N = 119)	N of patients	N of events	Hazard ratio	95 % CI	Wald’s p
Performance status					
1–2–3 vs. 0	32 vs. 87	27 vs. 61	*1.64*	1.03–2.62	0.036
TOP2A (FISH) ratio 2.0 copies 4.0					
Amplified vs. non-amplified	42 vs. 77	28 vs. 60	***0.62***	0.38–1.00	0.050
MYC CN					
Gain vs. no gain	10 vs. 109	10 vs. 78	*6.02*	2.67–13.6	<0.001
Type of disease*CEN profile (binary)					0.048
CEN profile, 1chr vs. other @ R-mBC	28 vs. 58	24 vs. 39	*2.29*	1.36–3.85	
CEN profile, 1chr vs. other @ de novo-mBC	12 vs. 21	9 vs. 16	0.77	0.29–2.04	
de novo-mBC vs. R-mBC @ CEN profile, other	21 vs. 58	16 vs. 39	1.15	0.64–2.08	
de novo-mBC vs. R-mBC @ CEN profile, 1chr	12 vs. 28	9 vs. 24	***0.39***	0.16–0.93	
HER2-positive (N = 83)					
TOP2A (FISH) ratio 2.0 copies 4.0					
Amplified vs. non-amplified	39 vs. 44	25 vs. 35	***0.58***	0.35–0.98	0.041
MYC CN					
Gain vs. no gain	7 vs. 76	7 vs. 53	*4.40*	1.90–10.2	0.001
HER2-negative (N = 45)					
Menopausal status					
Post vs. pre	28 vs. 17	25 vs. 10	*3.69*	1.57–8.65	0.003
ER/PgR					
Positive vs. negative	39 vs. 6	29 vs. 6	***0.22***	0.08–0.58	0.002
MYC FISH binary (ratio 2, copies >5)					
Amplified vs. non-amplified	4 vs. 41	4 vs. 31	*4.54*	1.40–14.70	0.012
Ki67 (by increase 5 %)			*1.16*	1.07–1.26	0.001
R-mBC (N = 84)					
Age					
≥50 vs. <50	56 vs. 28	39 vs. 23	***0.50***	0.28–0.89	0.019
Performance status					
1-2-3 vs. 0	25 vs. 59	22 vs. 40	*1.83*	1.07–3.14	0.028
ER/PgR					
Positive vs. negative	57 vs. 27	39 vs. 23	***0.43***	0.24–0.76	0.004
PI3K					
Activation vs. non-activation	74 vs. 10	56 vs. 6	*4.50*	1.63–12.39	0.004
MYC CN					
Gain vs. no gain	6 vs. 78	6 vs. 56	*20.48*	6.77–61.99	<0.001
de novo-mBC (N = 49)					
Performance status					
1-2-3 vs. 0	13 vs. 36	12 vs. 26	*1.99*	0.99–4.02	0.055
ER/PgR					
Positive vs. negative	36 vs. 13	27 vs. 11	***0.16***	0.03–0.77	0.023
HER2 status/subtypes					0.13
Luminal HER2 vs. HER2-negative	21 vs. 17	16 vs. 13	1.15	0.53–2.49	0.72
HER-enriched vs. HER2-negative	11 vs. 17	9 vs. 13	***0.20***	0.04–0.96	0.045

*N* number, *CI* confidence interval, *CN* copy number, *italics* unfavorable, *bold italics* favorable prediction

The outcome of patients with centrally evaluated HER2-negative tumors seemed significantly adversely affected by the absence of ER/PgR positivity, post-menopausal status, and by increased Ki67. In the group of 49 patients with de novo mBC, good performance status, ER/PgR positivity, and the HER2-Enriched subtype predicted for longer survival; in addition to these parameters, pre-menopausal status and MET amplification predicted for longer TTP. The statistically significant results obtained for MYC CN gain in this patient group, as well as for MYC amplification with FISH in HER2-negative patients, concerned only four patients in each case and were not considered to be clinically significant. Of note though, the four patients with MYC amplification also had increased CEN8 CN.

## Discussion

Discordant inter-laboratory HER2 status still represents an important issue in the clinic, even after more than 10 years of practicing and extensive external quality assurance rounds for method standardization and guideline updates on this predictive marker. The overall local vs. central testing discordance rate varies between 2.5 and 20 %, with HER2 heterogeneity and laboratory analytical and post-analytical issues as the major contributors to this discrepancy [32, 35–37]. More than twice as many discrepant cases are locally HER2-positive/centrally HER2-negative. Although some of these cases may be “true false positives”, accurate HER2 testing still represents a challenge [32, 37]. All above parameters and the fact that patients were treated with trastuzumab in the early years of HER2 testing may have accounted for the high discordance rate observed in the examined series upon retrospective central assessment. Whether the locally positive/centrally negative tumors were “false positives” can by no means be resolved; such tumors had nevertheless mean HER2 copy number of 3, indicating low level aberrations of the HER2 amplicon that might have contributed to HER2 over-expression. Further, patient outcome did not differ in the two groups. Hence, these tumors were considered to be of ambiguous HER2 status, as opposed to “true positives” upon local and central testing.

This study mainly focused on the effect of MYC and MET status on mBC patient outcome upon trastuzumab treatment and shows that MYC but not MET CN status adversely affected the outcome of mBC patients treated with trastuzumab. MYC and MET were assessed at multiple molecular levels, by multiple methods, all of which have intrinsic characteristics. At the gene level, FISH probes detect, although not exclusively, the gene of interest on the corresponding chromosome, while CN assessment with qPCR is gene specific but may suffer from PCR target efficiency and calibrator DNA characteristics; hence, the two methods do not always yield the same information on gene copy status [38]. By using the criteria for MYC amplification provided by Perez et al. [27], we recapitulated the findings of that study concerning the 15 % incidence of MYC amplification without any prognostic effect for this marker in HER2-positive patient, further supporting that co-amplified MYC and HER2 confer poor prognosis in patients treated with anthracyclines but not with anti-HER2 targeted drugs [39].

The incidence of tumors with MYC CN gain, as assessed with qPCR CNV assays, was only half compared to FISH MYC amplification; out of these cases, only half were concordant with both methods. In the only study to our knowledge comparing the two methods, conducted on medulloblastomas for MYC genes including MYC on chromosome 8 [40], FISH would detect less MYC amplified cases than qPCR. The opposite was noticed here. This implies that in some of the cases with FISH amplification, different aberrations than simply multiple MYC copies may exist, e.g., gene rearrangements in 8q24.21, a locus involved in established translocations in hematopoetic malignancies. Further, MYC was investigated here for mRNA and protein expression. Other than described in angiosarcomas, breast included [41], MYC gene status by either method in this study did not predict for MYC mRNA and protein over-expression, in line with previous observations in breast carcinomas [14]. Thus, overall, the novel finding concerning MYC in this study was the strongly unfavourable prognostic effect of CN gain in mBC patients treated with trastuzumab. Of note, MYC CN gain as a marker may be a method-limited surrogate, additionally biased by the small number of patients carrying this alteration. However, survival of all these 12 patients was shorter than the median observed for patients without MYC CN gain; thus, this marker may be considered for evaluation in larger series.

MET amplified calls with FISH and with qPCR practically hardly overlapped; tumors with MET CN gain by qPCR were ten times more than MET amplified tumors by FISH, while no association was observed between MET gene parameters and MET mRNA expression. Although the cut-off for FISH amplification was substantially lower than the one recently proposed [42], the rate of MET amplified cases in the present series was very low and their number too small for meaningful statistical analyses. The discrepancy between FISH and qPCR MET status was probably due to the low copy number load even in tumors with MET CN gains, as previously discussed [38, 40]. Nevertheless, MET CN gain showed a trend as an independent unfavourable marker in patients who presented with de novo mBC, which might be considered as a surrogate for an adverse effect of MET CN aberrations in the present clinical context.

An important piece of data from this study corresponds to the effect of CEN copy patterns in patients with trastuzumab treated mBC. Aberrant CEN copies under any name (polysomy, CEP-duplication, aneusomy) correspond to genomic/chromosonal instability in breast cancer [43, 44], may explain equivocal HER2 status [45] and may be related to tumor proliferation [44]. Herein, of the three centromeres studied, only increased CEN8 copies were associated with tumor proliferation. However, CEN profiles had diverse impact on patient outcome, according to HER2 status and presentation of disease, conferring opposite prognosis in patients with ambiguous HER2 as compared to true positives, as well as in patients with R-mBC as compared to de novo mBC. Importantly, normal or complex CEN gains had the same but opposite impact on prognosis as compared to single CEN gains, indicating that the extent of instability and not the chromosome target per se affects patient outcome. Chromosomal instability has so far been related to adjuvant anthracycline efficiency in breast cancer [43, 46]. The present study may be the first one to show a prognostic effect of chromosomal instability in the mBC setting involving trastuzumab treatment. This finding is important since it concerns about one-third of mBC patients treated with this drug, and mostly those with ambiguous HER2 status. However, whether the observed prognostic CEN profile effect is related to trastuzumab or to chemotherapy treatments that were also administered to these patients needs to be evaluated in appropriately designed prospective studies.

In addition, Ki67 labelling in a continuous mode reflecting tumor proliferation rate, and PI3K activation had distinct prognostic effects in mBC patient subsets in the present study. Tumor proliferation emerged as an independent poor prognosticator in patients with R-mBC but not with de novo mBC and in patients with ambiguous HER2 but not with true positive HER2 status. The latter may be related to the effect of increased CEN copies in the ambiguous HER2 group, linking proliferation with chromosomal instability, as previously reported [44]. Ambiguous and true HER2-positive tumors mainly differed in hormone receptor positivity and, probably because of that, in the incidence of PIK3CA mutations and PTEN loss. The latter were, as expected [47], more frequent in tumors with ambiguous HER2 status, which were also more frequently ER/PgR-positive. However, the presence of hormone receptors was the independent prognosis-relevant factor in both ambiguous and true HER2-positive patients. When examined separately, PIK3CA mutations, PTEN loss and mTOR activation were outcome-related in subsets of patients, but were not retained in multivariable models. However, all three parameters in combination, indicative of PI3K activation, were independently associated with poor prognosis in patients with R-mBC only. Although this finding may be biased by the small number of patients without PI3K activation in the present cohort, it appears to be in accordance with PI3K activation as a cause for trastuzumab resistance [48] and it also highlights the differences in disease biology in patients treated before the manifestation of metastases, as compared to those who present with metastatic disease at first diagnosis. Of note, primary tumors were examined in all cases of this study, but PI3K activation in those with de novo mBC did not seem to affect patient outcome. This may be important when considering PI3K inhibition as a therapeutic adjunct in mBC patients.

## Conclusions

The present study highlights the potential clinical relevance of MYC copy number status, PI3K activation and chromosomal instability in patients with mBC treated with trastuzumab for HER2-positive disease. These markers may have distinct impact on patient outcome according to solid or ambiguous positive HER2 status of the tumors, and to metastasis manifestation. Limitations of the study are its retrospective nature and the small size of the emerged patient subgroups with significant marker implications. If validated in studies with adequate statistical power, especially with regards to R-mBC and de novo mBC, the herein presented markers may prove important for the therapeutic assessment of patients with HER2-positive metastatic disease.

## Additional file

10.1186/s12967-016-0883-z Supplemental tables S1 to S10.

## Abbreviations

CEN: centromere
CN: copy number
DNA: deoxyribonucleic acid
ER: estrogen receptors
FFPE: formalin-fixed paraffin-embedded
FISH: fluorescence in situ hybridization
HER: human epidermal growth factor receptor
H&E: hematoxylin & eosin
IHC: immunohistochemistry
mBC: metastatic breast cancer
mTOR: mammalian target of rapamycin
PS: performance status
PgR: progesterone receptors
PI3K: phosphatidylinositol 3-kinase
qPCR: quantitative polymerase chain reaction
R-mBC: relapse from previously treated disease
RNA: ribonucleic acid
RQ: relative quantification
TCC: tumor cell content
TMA: tissue microarray
TOP2A: topoisomerase II Alpha
TTP: time to tumor progression
